# Loose Plant Architecture 1-Interacting Kinesin-like Protein KLP Promotes Rice Resistance to Sheath Blight Disease

**DOI:** 10.1186/s12284-021-00505-9

**Published:** 2021-07-02

**Authors:** Jin Chu, Han Xu, Hai Dong, Yuan Hu Xuan

**Affiliations:** 1 Institute of Plant Protection, Liaoning Academy of Agricultural Sciences, Shenyang, 110161 China; 2 College of Plant Protection, Shenyang Agricultural University, Shenyang, 110866 China

**Keywords:** KLP, Sheath blight disease, Transcription activation, Defense, Rice

## Abstract

**Background:**

Sheath blight disease (ShB) is a destructive disease affecting rice production. Previously, we have reported that Loose Plant Architecture 1 (LPA1) promotes resistance to ShB. However, the mechanisms by which LPA1 confers resistance against this disease have not been extensively investigated. Notably, interactors that regulate LPA-1 activity remain elusive.

**Findings:**

In this study, we identified the interaction of kinesin-like protein (KLP) with LPA1 in the nucleus of rice cells by yeast two-hybrid, bimolecular fluorescent complimentary (BiFC), and co-immunoprecipitation (co-IP) assays. To investigate the role of *KLP* in promoting resistance to ShB, wild-type, *klp* mutant, and *KLP* overexpressor (*KLP OX*) rice plants were inoculated with *Rhizoctonia solani* AG1-IA. The results indicated that, compared with the wild-type control, *klp* mutants were more susceptible while *KLP OX* plants were less susceptible to ShB. Since LPA1 transcriptionally activates *PIN-FORMED 1a (PIN1a)*, we examined the expression of 8 related *PIN* genes. The results showed that only the expression of *PIN1a* and *PIN3b* coincided with *KLP* expression levels. In addition, a chromatin immunoprecipitation (ChIP) assay showed that KLP bound directly to the promoter region of *PIN1a* but not of *PIN3b*. Transient expression assays confirmed that LPA1 and KLP transcriptionally activate *PIN1a*, and that coexpression of KLP and LPA1 had an additive effect on the activation of *PIN1a*, suggesting that KLP enhances LPA1 transcriptional activation activity.

**Conclusions:**

Taken together, our results show that KLP is a novel LPA1 interactor that promotes resistance of rice to ShB.

## Findings

*Rhizoctonia solani* (*R. solani)* is a causative agent of sheath blight disease (ShB) in rice (*Oryza sativa*) that severely affects rice production in China (Savary et al. 1995). Damage inflicted by ShB occurs during the entire rice cultivating period, and mainly affects the leaves, sheaths, and panicles (Savary et al. 1995). When the disease is severe ShB reduces the yield by up to 50% (Savary et al. 2000). Nowadays, fungicide application is the main approach to control ShB, due to a lack of resistant cultivars and resistance-related genes (Savary et al. 2000). However, the use of pesticides results in severe pollution and increases the cost of cultivation. Therefore, there is an urgent need to identify resistance-related genes and to use those genes to obtain resistant rice cultivars to protect rice from ShB.

Extensive studies have shown that overexpression of chitinase, β-1,3-glucanase, and polygalacturonase inhibiting protein1 (OsPGIP1) could enhance the resistance of rice to *R. solani* (Shah et al. 2009; Mao et al. 2014; Wang et al. 2015). Inducible expression of OsACS2, an 1-aminocyclopropane-1-carboxylic acid (ACC) synthetase that is a key enzyme in ethylene synthesis, promotes rice resistance to blast and sheath blight (Helliwell et al. 2013). Overexpression of BROAD-SPECTRUM RESISTANCE2 (BSR2) has been shown to increase rice resistance to *R. solani* (Maeda et al. 2019). Salicylic acid-triggered defense mechanisms play an important role in resistance to *R. solani* (Kouzai et al. 2018). OsARS2, Os2H16, and OsGSTU5 are positive regulators of resistance of rice to ShB (Tiwari et al. 2020; Li et al. 2018), while OsARS2 directly regulates Os2H16 via binding of a GT1 cis-element in the promoter region (Li et al. 2018). A genome-wide association study identified the F-box protein ZmFBL41 as a negative regulator of the resistance of maize to banded leaf and sheath blight through its interaction with ZmCAD, a monolignol biosynthesis enzyme. The rice homologous gene *OsCAD8b* plays a similar function in the defense against ShB (Li et al. 2019). Our recent work demonstrated that the sugar transporter 11 (*SWEET11*) negatively regulates the defense of rice against ShB (Gao et al. 2018), while the transcription factor DOF11 activates *SWEET14* promoting resistance of rice to ShB (Kim et al. 2020). This is related to ABI3/VP1-Like 1 (RAVL1) that positively regulates the defense of rice against ShB by modulation of brassinosteroids and ethylene signaling (Yuan et al. 2018). Overexpression of *Loose Plant Architecture 1* (*LPA1*), containing an indeterminate domain (IDD), promoted the defense of rice against ShB via activation of *PIN1a* (Sun et al. 2019). Furthermore, IDD13, IDD3, and the G-protein γ subunit DEP1 interact with LPA1 to differentially regulate the resistance of rice to ShB (Miao Liu et al. 2020; Sun et al. 2020). However, the mechanism by which LPA1 regulates resistance against ShB remains to be investigated.

**Fig. 1:**
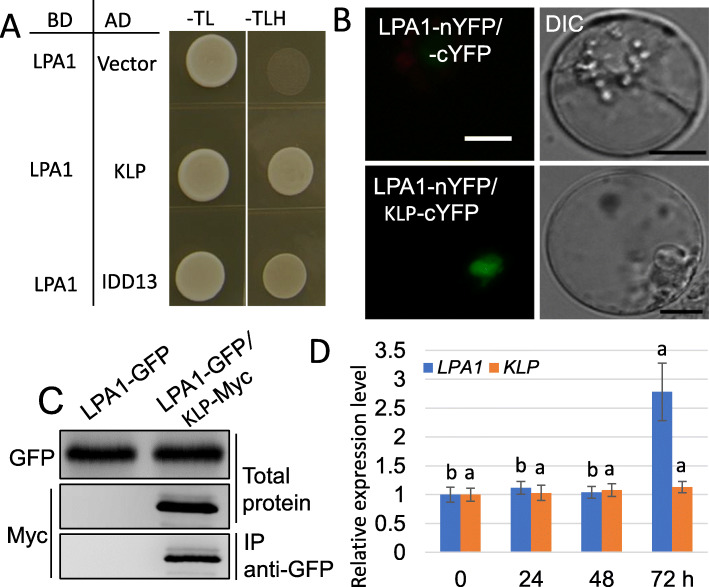
Interaction between KLP and LPA1. **a** The interaction between LPA1 and KLP or IDD13 was analyzed by yeast two hybrid (Y2H) assay. BD: GAL4-DNA binding domain; AD: activation domain; −TL: SD medium without tryptophan and leucine; −TLH: SD medium without tryptophan, leucine, and histidine. **b** LPA1-nYFP + − cYFP or LPA1-nYFP + KLP-cYFP were coexpressed in rice protoplasts to detect YFP protein reconstruction. Bars = 10 μm. **c** The interaction between LPA1 and KLP was analyzed in tobacco leaves by co-IP. LPA1-GFP+ KLP-Myc or LPA1-GFP were transformed into tobacco leaves using Agrobacterium-mediated transformation. Western blot analysis used an anti-Myc or anti-GFP antibody. Anti-GFP antibody was used to immunoprecipitation. **d** Relative expression patterns of *LPA1* and *KLP* were examined at 0, 24, 48, and 72 h post-inoculation (hpi) with *R. solani* AG1-IA. The error bars indicate the mean ± SE (*n* = 3). Different letters indicate significant differences at *P < 0.05*

To investigate the mechanism by which LPA1 regulates the resistance of rice to ShB, we performed a yeast two-hybrid (Y2H) screen. Among potential LPA1 interactors, we identified a kinesin-like protein (KLP). The Y2H results indicated that LPA1 interacts with KLP and IDD13 (Fig. 1a). Furthermore, a split-GFP assay was performed in rice protoplasts, confirming that LPA1 interacts with KLP in the nucleus, while no visible signal was detected in the negative control (LPA1-nYFP+cYFP) (Fig. 1b). In addition, a co-IP was carried out where KLP-Myc was coexpressed with LPA1-GFP in *N. benthamiana* leaves, and an anti-GFP antibody was used to immunoprecipitate LPA1-GFP. Western blot analysis using an anti-Myc or anti-GFP antibody indicated that KLP-Myc and LPA1-GFP were successfully expressed and that LPA1 also interacts with KLP in plants (Fig. 1c). Since *LPA1* expression was induced by inoculation of *R. solani*, we also examined *KLP* expression upon inoculation with *R. solani*. qRT-PCR data showed that *LPA1* was induced after 72 h of the inoculation, but *R. solani* inoculation did not change the expression levels of *KLP* (Fig. 1d).

**Fig. 2:**
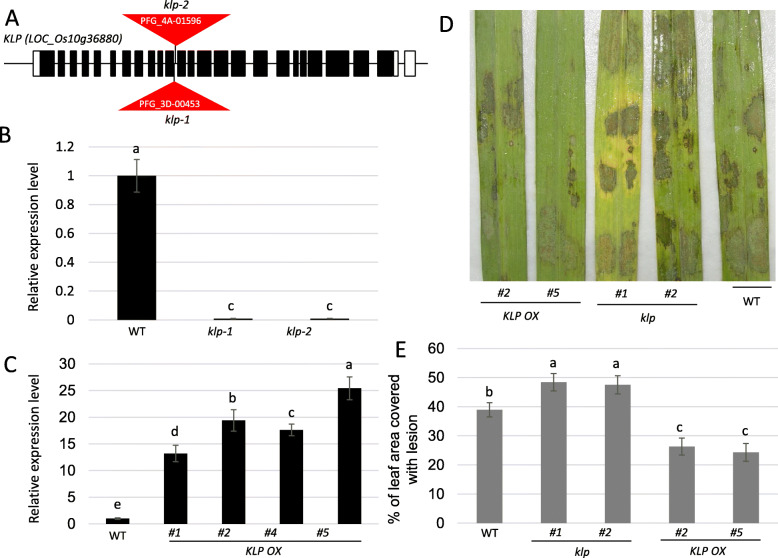
KLP promotes resistance of rice to ShB. **a** Genomic structure of *KLP* mutants. White and black boxes indicate untranslated regions (UTR) and open reading frame (ORF) region, respectively. The lines between black boxes indicate introns. The red triangles indicate T-DNA insertion sites. The labels inside triangle indicate mutant numbers from SALK (http://signal.salk.edu/cgi-bin/RiceGE). *klp-1* and *klp-2* are the individual *KLP* insertional mutants. **b** Expression levels of *KLP* in wild-type (WT) and *KLP* mutants (*klp-1* and *klp-2*). The error bars indicate the mean ± SE (*n* = 3). Different letters indicate significant differences at *P < 0.01*. **c** Expression level of *KLP* was analyzed in WT and *KLP* overexpressors (*OX #1*, *#2*, *#4*, and *#5*). The error bars indicate the mean ± SE (*n* = 3). Different letters indicate significant differences at *P < 0.05*. **d** Wild-type (WT), *klp* mutants (*#1* and *#2*) and *KLP OX* (*#2* and *#5*) plants were inoculated with *R. solani* AG1-IA. **e** Percentage of leaf area covered with lesions in the plant lines shown in (d). Data represent the means ± standard error (*n* > 15). The error bars indicate the mean ± SE (*n* = 3). Different letters indicate significant differences at *P < 0.05*

To analyze the role of *KLP* in promoting resistance of rice to ShB, *klp* mutants and *KLP* overexpression lines were generated. Two independent *klp* mutants named *klp-1* and *klp-2*, were generated by insertion of T-DNAs into the 11th intron (Fig. 2a). qRT-PCR data showed that no *KLP* transcripts were detected in *klp-1* and *klp-2* mutant plants (Fig. 2b). In parallel, the *KLP* expression level was examined in wild-type and 4 *KLP* overexpressors (*KLP OX*) lines (*#1*, *#2*, *#3*, and *#5*). The qRT-PCR data showed that *KLP* expression levels were higher in *KLP1 OXs* compared with wild-type plants, and the highest expression was detected in *KLP OX #5* (Fig. 2c). Inoculation with *R. solani* AG1-IA revealed that, compared with wild-type plants, *klp* mutants (*klp-1* and *klp-2*) were more susceptible (*p* < 0.05) while *KLP OX* plants (*#2* and *#5*) were less susceptible (*p* < 0.05) to ShB (Fig. 2d). The percentage of the leaf area covered with lesions was 39.1% in WT, 48.2% in *klp-1*, 47.2% in *klp-2*, 27.5% in *KLP OX #2*, and 26.5% in *KLP OX #5* plants (Fig. 2e).

**Fig. 3:**
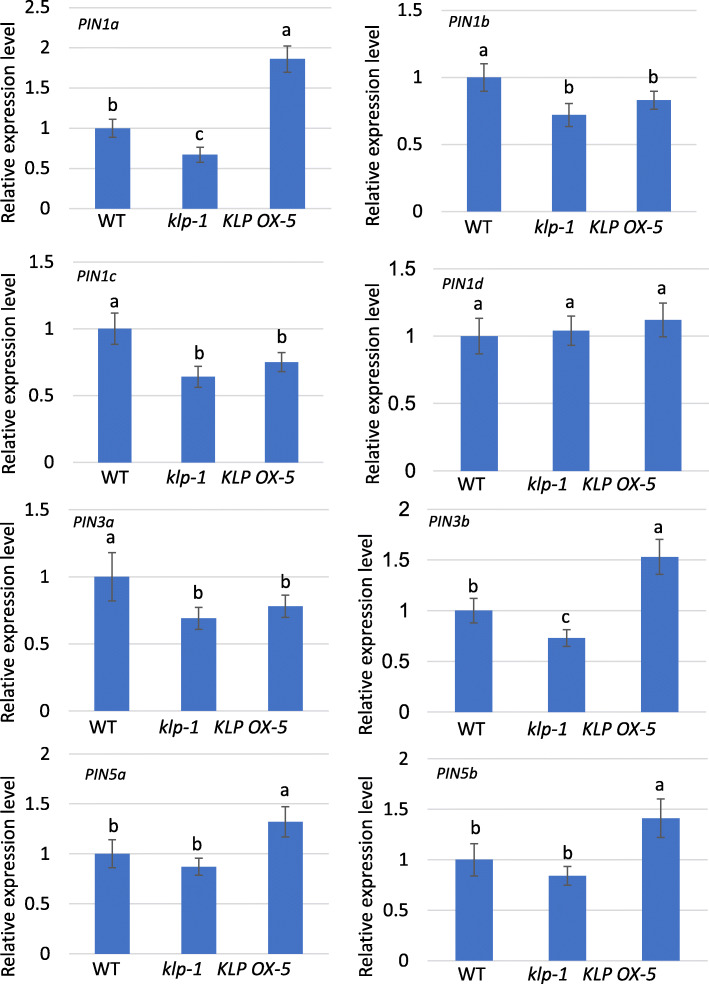
Expression levels of *PIN* genes in wild-type, *klp*, and *KLP OX* plants. Shown are the expression levels of *PIN1a*, *PIN1b*, *PIN1c*, *PIN1d*, *PIN3a*, *PIN3b*, *PIN5a*, and *PIN5b* in one-month-old, *klp-1* and *KLP OX-5* plant leaves relative to WT plants. The error bars indicate the mean ± SE (*n* = 3). Different letters indicate significant differences at *P < 0.05*

Previously, we have found that LPA1 regulates the resistance of rice to ShB by directly activating *PIN1a* expression. To test whether KLP also regulates *PIN* gene expression, the expression levels of 8 *PIN* genes were analyzed in wild-type, *klp-1*, and *KLP OX-5* plants. The results showed that *PIN1a* and *PIN3b* expression levels were suppressed in *klp-1* and had increased in *KLP OX-5* plants compared to wild-type plants. *PIN1b*, *PIN1c*, and *PIN3a* expression levels were suppressed in both *klp-1* and *KLP OX-5* plants compared to wild-type plants. *PIN5a* and *PIN5b* expression levels were higher in *KLP OX-5* compared to wild-type plants, while no differences in *PIN5a* and *PIN5b* expression levels were observed between wild-type and *klp-1 plants*. Meanwhile, the expression level of *PIN1d* was similar between wild-type, *klp-1*, and *KLP OX-5* plants (Fig. 3).

**Fig. 4:**
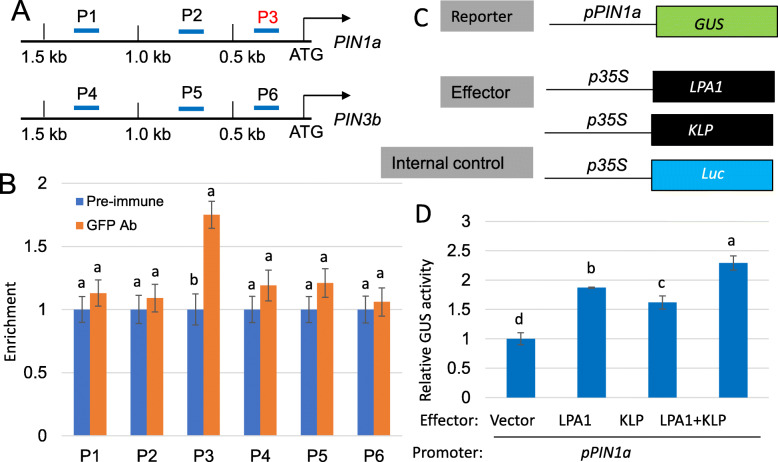
LPA1 and KLP directly activate *PIN1a*. **a** Schematic diagram showing the location of the probes (P1-P3 and P4-P6) used for chromatin immunoprecipitation (ChIP) assay within the 1.5 kb promoter regions of *PIN1a* and *PIN3b*, respectively. **b** The DNA fragments were immunoprecipitated from *p35S:KLP:GFP* transgenic plants calli, and the enrichment was analyzed by qPCR. Input DNA was used to normalize the data. Anti-GFP antibody was used for immunoprecipitation with pre-immune serum as control. Error bars represent the mean ± SE (*n* = 3). Different letters indicate significant differences at *P < 0.05*. **c** Schematic diagram indicating the constructs used in the transient assay. 1.5 kb of *PIN1a* promoter was used to drive *ß-glucuronidase* (*GUS*) gene coding sequences. 35S promoter was used to drive *LPA1*, *KLP* or *luciferase* (*Luc*) gene OFR sequences. **d** Plasmids corresponding to *p35S:LPA1*, *p35S:KLP*, *p35S:KLP + p35S:LPA1* were co-transformed with the vector expressing the *GUS* under the control of the *PIN1a* promoter (*pPIN1a*) in protoplasts. The luciferase expression level was utilized to normalize the GUS expression. Error bars represent the mean ± SE (*n* = 3). Different letters indicate significant differences at *P < 0.05*

Since *PIN1a* and *PIN3b* expression was positively regulated by KLP, the affinity of KLP to *PIN1a* and *PIN3b* promoters was examined. Three regions within the 1.5 kb promoter regions of *PIN1a* (P1-P3) and *PIN3b* (P4-P6), respectively (Fig. 4a), were tested by ChIP PCR using *KLP-GFP* transgenic plants. The immunoprecipitation was performed using the pre-immune (control) and anti-GFP antiserum. The ChIP-PCR results showed that KLP directly bound to the P3 region of the *PIN1a* promoter, but no binding affinity was observed in *PIN3b* promoter region (Fig. 4b). To verify that LPA1 and KLP bind to the P3 region of the *PIN1a* promoter and activate its expression, transient expression assays were performed using rice protoplasts. The *35S:LPA1*, *35S:KLP*, or *35S:LPA1* + *35S:KLP* plasmids were cotransformed with a construct expressing the *ß-glucuronidase* gene (*GUS*) under the control of the 1.5 kb *pPIN1a* promoter in the protoplasts. A *35S:Luc* (*luciferase*) plasmid was used as the internal control for evaluation of transformation efficiency (Fig. 4c). Transient assay results showed that LPA1 and KLP activated *pPIN1a*, and that LPA1 had a higher *pPIN1a* activation activity than KLP. Interestingly, coexpression of LPA1 and KLP resulted in a stronger transcriptional activation of *pPIN1a* than expression of either LPA1 or KLP alone (Fig. 4d), indicating an additive effect of KLP on LPA1-mediated activation of *pPIN1a*.

The isolation of resistance-related genes and the breeding of rice plants using these genes is the most efficient way to control disease-mediated loss in rice production. ShB is a destructive rice disease that causes severe yield reduction. However, the molecular mechanism remains to be determined. Previously, we reported that the IDD-containing protein LPA1 promotes resistance to ShB. In the current study, we have shown that KLP interacts with LPA1 in the nucleus, which was verified by yeast two-hybrid, split-GFP, and co-IP assays. Further genetic analysis using inoculation of *KLP* mutants and overexpressing plants with of *R. solani* AG1-IA strain revealed that KLP promotes rice resistance to ShB. Two independent alleles of *klp* mutants were more susceptible while two *KLP OXs* were more resistant to ShB compared to wild-type plants. These results suggest that KLP is an LPA1-interacting protein that positively regulates the defense of rice against ShB. Furthermore, our qPCR results demonstrated that *PIN1a* and *PIN3b* expression levels positively correlated with *KLP* levels, while the expression of other *PIN* genes was differentially regulated by KLP. A ChIP assay using *KLP-GFP* transgenic plants revealed that KLP directly bound to the *PIN1a* but not to the *PIN3b* promoter region. It has been previously reported that the kinesin-like protein BRITTLE CULM12 (BC12) directly binds to the *KO2* promoter of the gibberellic acid (GA) biosynthesis gene directly regulating its expression (Li et al. 2011), indicating that a KLP-type protein can function as a transcriptional regulator. Further transient assays confirmed that KLP and LPA1 activate a 1.5 kb fragment containing the *PIN1a* promoter, and KLP plays an additive function in LPA1-mediated *PIN1a* activation. *KLP* is not transcriptionally activated by infection of *R. solani*, implying that KLP-mediated rice resistance to ShB might be through activation of downstream gene expressions. *PIN1a* is a polar auxin transporter, and genetic studies have revealed that *PIN1a* positively regulates the defense mechanism against ShB in rice. Ethylene functions as positive or negative regulator of plant immunity depends on the type of pathogen, and auxin generally thought of as negative regulator of plant immunity (Yang et al. 2013). Also, ethylene and auxin play opposite role in rice defense to blast disease (Yang et al. 2013), however, exogenous treatment of auxin or activation of ethylene signaling promotes the resistance of rice to ShB (Yuan et al. 2018; Sun et al. 2019), suggesting that auxin and ethylene all play positive role in rice defense to ShB. Also, KLP might regulate *PIN1a* transcription to modulate local auxin content resulting in increased resistance.

In conclusion, we have shown that KLP, a kinesin-like protein, interacts with transcription factor LPA1 to activate downstream gene expression in a dosage-dependent manner. Our analyses demonstrated that KLP and LPA1 together directly activate *PIN1a* expression. PIN1a is an ortholog of AtPIN1a, which may control auxin transport to modulate auxin distribution (Petrasek and Friml 2009), and the increase of local auxin concentration promotes resistance of rice to ShB (Sun et al. 2019). Taken together, our results suggest that KLP partners with LPA1, to promote resistance rice to ShB via activation of PINa-dependent auxin redistribution and subsequent activation of auxin signaling.

## Data Availability

The datasets supporting the conclusions of this article are provided within the article and its additional files.

## Abbreviations

KLP: Kinesin-like protein
IDD: Indeterminate domain
ShB: Sheath blight disease
PGIP1: Polygalacturonase inhibiting protein
PIN: PIN-FORMED
LPA1: Loose plant architecture1
SWEET11: Sugar will be eventually exported transporter
RAVL1: Related to ABI3/VP1-Like 1
WT: Wild type
OX: Overexpressor
ChIP: Chromatin-immunoprecipitation
Co-IP: Co-immunoprecipitation
BC12: BRITTLE CULM12
GA: Gibberellic acid
GUS: ß-glucuronidase
Luc: Luciferase
Y2H: Yeast two-hybrid
